# Responses and reliability of candidate intensity measures to different mental and motor load levels of an upper limb exergame in children and adolescents with neurological diagnoses

**DOI:** 10.3389/fresc.2025.1641003

**Published:** 2025-10-20

**Authors:** Gaizka Goikoetxea-Sotelo, Hubertus J. A. van Hedel

**Affiliations:** ^1^ Swiss Children’s Rehab, University Children’s Hospital Zurich, University of Zurich, Affoltern am Albis, Switzerland; ^2^ Children’s Research Center, University Children’s Hospital Zurich, University of Zurich, Zurich, Switzerland

**Keywords:** intensity measures, neurorehabilitation, movement repetitions, heart rate variability, activity counts, skin conductance, perceived effort, NASA-TLX

## Abstract

**Background:**

High-intensity therapy improves outcomes in (pediatric) neurorehabilitation, yet standardized intensity measures accounting for motor and/or mental demands remain scarce.

**Objectives:**

To evaluate the responses and test-retest reliability of heart rate variability (HRV), skin conductance (SC), activity counts and movement repetitions normalized for the maximal capacity (%ACmax and %MOVmax, respectively), and the NASA-TLX across personalized motor and mental load levels in children and adolescents with neurological diagnoses using upper limb exergames.

**Methods:**

In a cross-sectional study, participants engaged in two custom exergames at three intensity levels (“very easy,” “challenging,” “very difficult”), each lasting 3 min. Responses of the candidate intensity measures were analyzed across conditions, and intraclass correlation coefficients (ICC) assessed reliability across two consecutive sessions.

**Results:**

30 children and adolescents with neurological diagnoses aged 9–19 years participated in the study. %MOVmax and NASA-TLX (overall, effort, mental) responded to both mental and motor intensity increases. HRV, %ACmax, and NASA-TLX physical subscale responded to motor load only. SC showed no consistent response. HRV and %ACmax demonstrated the highest reliability (ICC > 0.75), especially under motor conditions. NASA-TLX effort showed potential as a simplified surrogate for the full scale, though with variable reliability.

**Conclusion:**

Changes in motor intensity were better captured than changes in mental intensity. Combining HRV, %ACmax, and NASA-TLX effort could offer a multidimensional approach to quantify therapy intensity. However, many measures lacked sufficient reliability or feasibility for clinical implementation. Further research is needed to validate these measures in real-world therapeutic settings and clarify their relationship to individual capacity.

## 1 Introduction

The primary aim of (pediatric) neurorehabilitation is to enhance the patient's independence and quality of life (1, 2). The various therapies a patient receives are individually and dynamically tailored to the patient's situation, abilities, and goals, following motor learning theories (3). To monitor progress and guide treatment, treatment effectiveness is evaluated using standardized outcome measures. Among the different factors influencing these outcomes, therapy intensity has emerged as a key determinant of rehabilitation success (4).

Page and colleagues (5) defined intensity as “the amount of mental or motor work put forth by a patient during a particular movement or series of movements, exercise, or activity during a defined period of time”. This definition acknowledges the multidimensional nature of intensity. We recommended to limit the defined period to a single therapy session (6), since, according to the general adaptation syndrome and periodization theories (7, 8), long-lasting changes in the system result from repeating sessions of sufficient intensity, but not excessive, over time.

Because therapy intensity is a key determinant of rehabilitative success (4), assessing it is essential. Reliable quantification is needed not only to monitor progress, but also to compare interventions, which requires balancing therapies for total dose. As dose includes both duration and intensity (6), intensity must be measured alongside time. Finally, a measure that can be applied continuously during therapy sessions would enable clinicians to adapt intensity in real time to the patient's physical condition.

Despite therapy intensity being one of the most important elements influencing rehabilitation outcomes, no standardized and generally accepted method exists for quantifying it (6). Because of its simplicity, therapy intensity is often measured as the time a patient spends in therapy. However, time spent in therapy is a bad indicator of a patient's (active) contribution to therapy (9). For example, a patient can perform 10 or 100 repetitions of a desired movement during a 30 min therapy session, but the time does not account for that. Time spent in therapy also does not consider the motor complexity and mental load required by the task. For instance, when practicing in-hand object manipulation, the therapist could increase the exercise load by asking the patient to manipulate smaller objects. This requires enhanced fine motor performance, motor planning, and concentration, leading to increased intensity. These requirements likely lead to slower and more precise movements, and quantifying intensity by measuring only the amount of movement is inappropriate.

Furthermore, an intensity measure should be relative, i.e., it should assess the patient's contribution relative to their capacities. Although absolute measures like activity counts (AC) or number of movement repetitions (MOV) have been demonstrated to be more accurate indicators of a patient's active engagement than time spent in therapy (10, 11), they do not consider the patient's abilities. For instance, while a patient with moderate impairments can perform 100 repetitions of a certain movement in ten minutes when working at their maximum capacity, another patient with severe impairments may be able to complete only ten repetitions in these ten minutes when working at their maximum capacity. Nevertheless, obtaining additional information, such as the maximum achievable counts (ACmax) or movement repetitions (MOVmax) a patient can complete for a particular exercise and time frame, enables to estimate the relative intensity level (%ACmax or %MOVmax) at which the patient works (12).

Self-reported effort scales, like the NASA-TLX, measure the relative exercise intensity the patients work at while providing a more compound and multidimensional picture (13). Furthermore, the questionnaire has been proven to differentiate between difficulty levels and task demands (14, 15) in a simple, sensitive, and valid manner (16, 17). Nevertheless, self-reported effort scales may not be the best tool to measure therapy intensity online, as constantly posing questions could disturb the patient and compromise the therapy session.

Physiological measures like heart rate variability (HRV) and skin conductance (SC) offer objective insights into heart-brain interaction and the modulation of the central nervous system (18, 19). Given their properties, research on healthy adolescents and adults has observed HRV and SC changes in response to increasing mental and motor load (20–22), suggesting their potential utility for measuring exercise intensity in pediatric upper limb neurorehabilitation.

Even though all the measures we just mentioned show potential for measuring exercise intensity, their response to variations in therapy intensity, particularly among children, remains largely unexplored. Our previous publications (12, 23) provided valuable insights on the responses of HRV, SC, AC, MOV, and perceived effort scales to changes in exercise intensity. However, neither study provided definitive evidence for clinical practice. Although one study (23) replicated a clinical environment, it did not control for motor and mental load, limiting interpretability. The other study (12) controlled for motor and mental load, but included only typically developing children, leaving unanswered whether these measures are responsive and reliable in children with neurological diagnoses, the very population that would benefit the most from such measures.

Therefore, in this study, we investigated the ability of HRV, SC, %ACmax, %MOVmax, and NASA-TLX to respond to different levels of personalized motor and mental load during exergame-based therapy in children with neurological diagnoses. We hypothesized that 1) HRV would decrease, whereas the SC and NASA-TLX scores would increase with increasing mental and motor demands, and 2) %ACmax and %MOVmax would increase with increased motor demands but remain unaffected by increased mental demands. Furthermore, we examined their test-retest reliability, as reliable measures are essential before translation to clinical practice.

By identifying measures that respond to different load levels and are reliable, this study aims to provide a foundation for standardizing the quantification of therapy intensity. Such standardization could enable more precise tailoring of interventions, greater comparability across studies and clinical programs, and, ultimately, improved rehabilitation outcomes for children with neurological diagnoses.

## 2 Methods

### 2.1 Participants

Following COSMIN guidelines (24), which suggest that 30 participants is the minimum for psychometric studies, our goal was to recruit 30 children and adolescents. Furthermore, we performed a sample size calculation based on HRV data from our previous study (12), focusing on the smallest observed difference (between the “challenging” and “very difficult” motor intensities). Imputing the effect size (f = 0.703) in *wp.rmanova()* (RStudio Inc., Boston, USA), the required sample size for 90% power (*β* = 0.10, *α* = 0.05) was estimated at 27.16 participants, which, accounting for 2–3 dropouts or data loss justifies the choice of 30. Inclusion criteria were the following: age 5–21 years, neurological conditions affecting their upper limbs, ability to understand and follow easy instructions, ability to communicate discomfort or pain. Exclusion criteria were the following: presence of screen-triggered epilepsy, skin lesions or conditions on the locations where we had to position the sensors for measuring the heart rate or SC, and the use of medication affecting central nervous system reactions (e.g., statins). We derived information on the age, sex, diagnosis, weight, and height from the electronic patient records.

We informed the potential participants and their legal representative(s) verbally about the study and, for those aged ten years and older, also in writing. Assent was mandatory from all participants and their legal representative(s). We also obtained written consent from at least one of the legal representatives and children older than 13 years of age. The Ethics Committee of the Canton of Zurich approved the study (BASEC no. Req-2021-01373). We performed the study following the Declaration of Helsinki and good clinical practice guidelines.

### 2.2 Procedures

#### 2.2.1 General procedures

**Figure 1:**
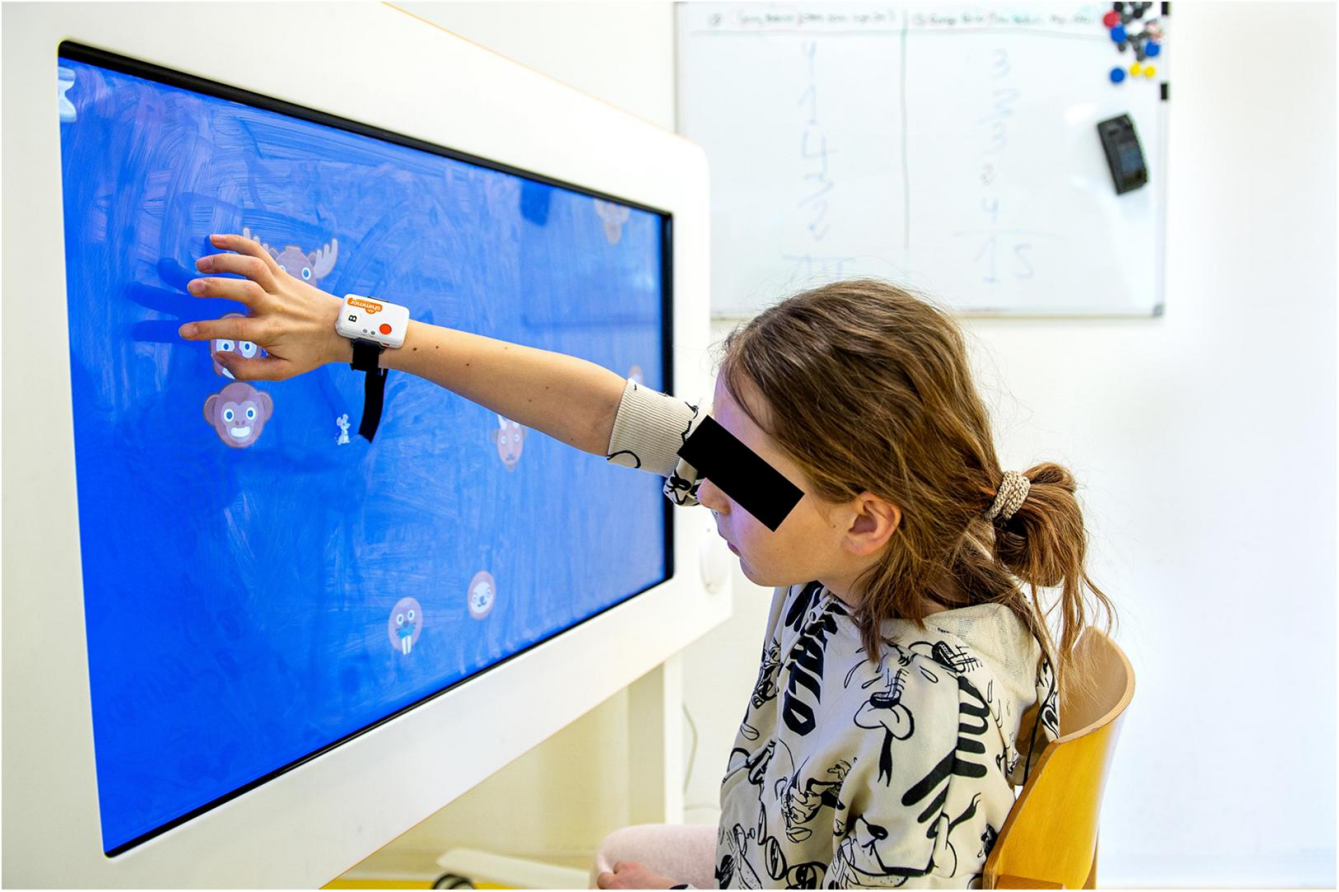
A child plays the custom-made mental exergame using the Myro® while wearing a Shimmer® inertial measurement unit (IMU) at the wrist. The Myro® is a device with a 941 × 529 mm touchscreen. The therapist can adapt the device to the patient's needs by adjusting the angulation, height, and work surface. The device responds to motion, pressure, and pulling, and patients can steer the games using their hands or objects that require different grasps. In therapy, the Myro® enables the training of gross and fine motor skills through video gaming. For this study, children steered the game using their dominant hand and fingers. Reprinted with permission from (11), licensed under CC BY, https://doi.org/10.1371/journal.pone.0326371.

We conducted the study at the Swiss Children's Rehab of the University Children's Hospital Zurich between December 2023 and February 2025. During two 60-minute sessions in two consecutive days, the participants played two custom-made exergames (the mental and the motor exergame) on the Myro® (Tyromotion, Graz, Austria; see Figure 1). The exergames were specifically designed to investigate the responses of candidate intensity measures to different levels of mental and motor load. Their practicability and appropriateness have already been tested in typically developing children and adolescents (12). The *mental exergame* is a visual search task in which participants have six seconds to locate and hit a target among distractors using a mouse and their less affected hand. The number of distractors depends on the intensity level (i.e., the higher the intensity, the more the distractors) and the individual participant's ability, and is determined by a calibration test. The *motor exergame* requires gross motor arm movements to hit a balloon within a given time, using the more affected hand. The time depends on the intensity level (i.e., the higher the intensity, the shorter the time), which is determined by a calibration test that accounts for the individual participant's ability.

Participants engaged in each exergame at three personalized difficulty levels (“very easy,” “challenging,” and “very difficult”), defined through a calibration procedure. In the *mental exergame* (visual search), distractors were incrementally added until the participant failed three consecutive trials; the highest successful level defined the calibration output, from which intensity levels were set at 50%, 100%, and 150% (adjusted for child-friendliness). In the *motor exergame* (balloon-hitting), participants completed a 30 s test where balloons reappeared within 60%–80% of their range of motion; the mean hitting speed defined the calibration output, and intensity levels were set to 30%, 70%, and 100%, following rehabilitation game design principles.

To account for fatigue, we randomized the order of appearance of the intensity levels and exergames. The measurement was repeated on two consecutive days to assess the test-retest reliability of the measures. To account for daily form and learning effects, we repeated the calibration tests each measurement day. A more detailed explanation of the protocol and the exergames can be found in Goikoetxea-Sotelo & Van Hedel (12). The protocols only differ in the mental condition, where participants played the exergame using a mouse instead of their hand to reduce the motor load.

**Figure 2:**
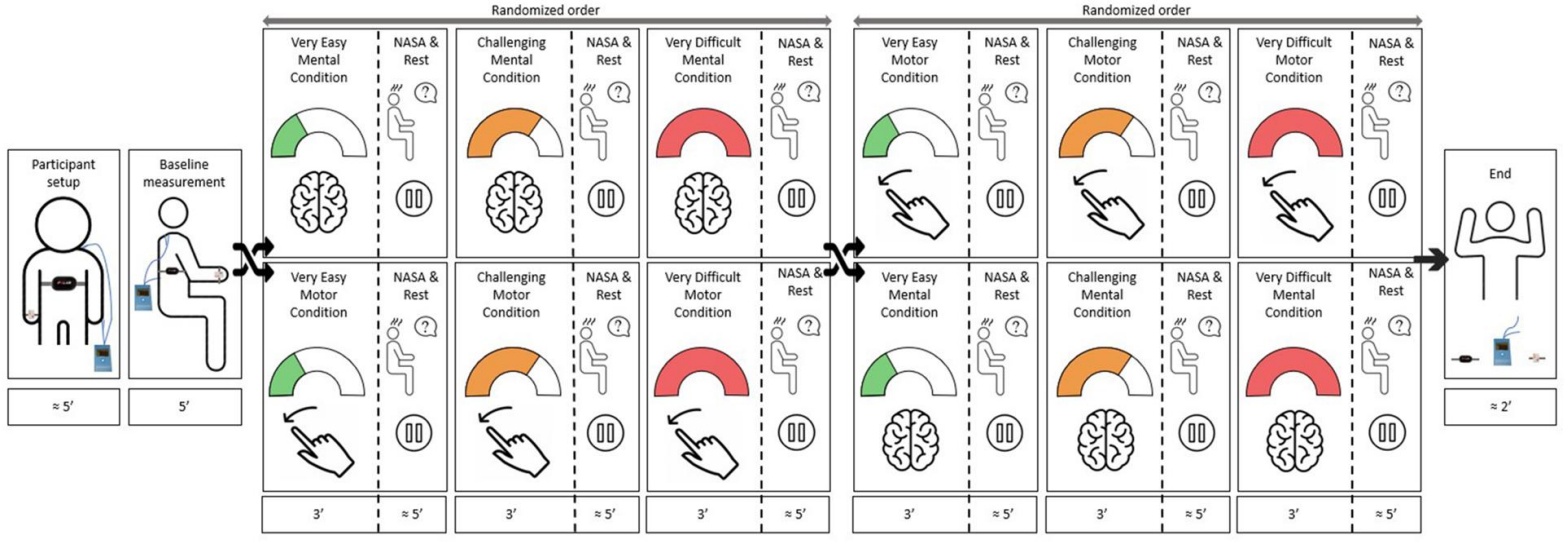
Study protocol. After preparing the participant and performing a baseline measurement, the participant played the two exergames in a randomized order. Each exergame consisted of a familiarization, a calibration, and a measurement, where the participant played each exergame at three intensity levels (i.e., “very easy”, “challenging”, and “very difficult”) in a randomized order. While playing, we recorded the responses of the various intensity measures to each intensity level. After each intensity level, the participant answered the NASA-TLX questionnaire. We repeated the protocol on two consecutive days to assess the test-retest reliability.

**Figure 3:**
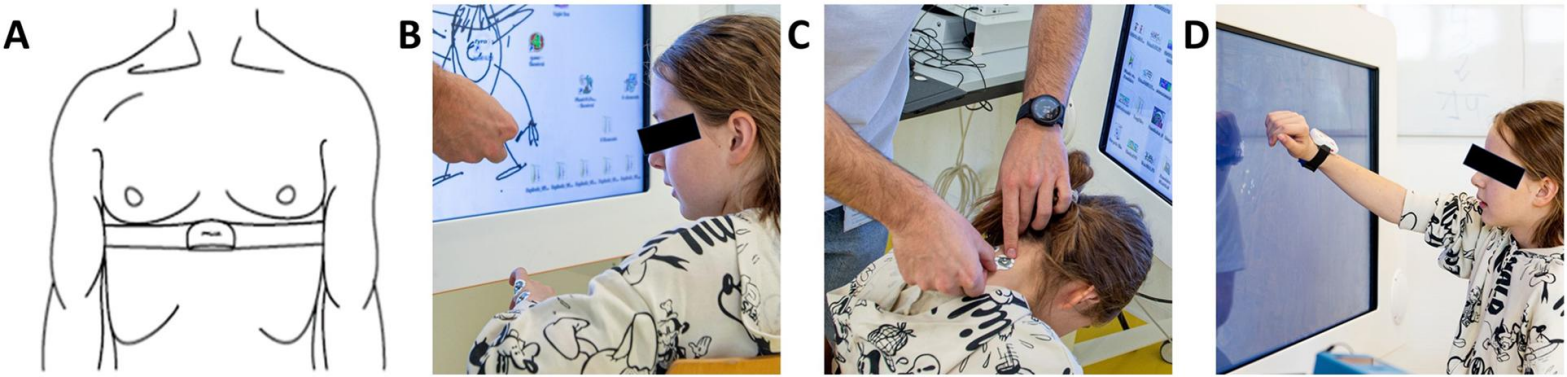
Participant setup. **(A)** A chest strap with a Polar H10 Heart Rate Sensor tied tightly under the pectoral muscles measures heart rate variability. We assessed skin conductance using electrodes positioned on **(B)** the hypothenar side of the non-dominant hand and **(C)** the neck para-medial below the hairline. **(D)** An IMU sensor placed dorsally on the wrist of the dominant hand measures activity counts. The blue box on the table is the MentalBioScreen K3 device for measuring skin conductance. Reprinted with permission from (11), licensed under CC BY, https://doi.org/10.1371/journal.pone.0326371.

After welcoming the participant, we explained the procedures and protocols and attached the sensors (Figures 2, 3). We ensured each participant could reach all playable areas by customizing the workspace to their unique range of motion (ROM). The protocol included three phases per exergame: familiarization, calibration, and measurement. In the familiarization phase, we explained the tasks and aims of the exergames while the participant played them for two minutes. In addition, we introduced the NASA-TLX, clarified its components to reduce measurement error, and asked the participant to contemplate nuanced responses instead of relying solely on extremes (25). In the calibration phase, we conducted a maximal capacity test to tailor the intensity levels to each participant's capabilities. In the measurement phase, the participant engaged with each intensity level for 3 min. At the end of each level, the participant responded to the NASA-TLX questionnaire.

### 2.3 Success rate and candidate intensity measures, including data processing

#### 2.3.1 Game-based control variable: success rate

The success rate, the control variable that helps us understand whether we set the intensity levels as intended, describes the percentage of correct interactions with the exergame [i.e., 100·(correct interactions/correct + incorrect interactions)].

#### 2.3.2 Physiological measures

##### 2.3.2.1 Heart rate variability

Heart rate variability was computed from heart rate data collected with the Polar H10 Heart Rate Sensor at 1,000 Hz (Polar Electro Oy, Kempele, Finland) (Figure 3A) and stored in the Elite HRV application (Asheville, USA). We processed the raw data using Kubios HRV Standard 3.5.0 (University of Eastern Finland/Kubios Oy, Finland) and performed additional data mining in MATLAB® Runtime R2025a (MathWorks, Natick, USA). To correct artifacts, we applied a manual filter-based threshold allowing interpolation of up to 1% of erroneous R–R intervals (26–28). We selected manual filtering over “automatic” (29) or “strong” (26) filters, which tend to over remove extreme intervals and thereby underestimate HRV. Subsequent signal processing was performed automatically in Kubios, including detrending (removal of frequencies <0.04 Hz), cubic spline interpolation, and 4 Hz resampling (30). Finally, we normalized the time-domain data by dividing it by the mean R–R interval (31, 32), which corrects for the nonlinear inverse relationship between R–R intervals and heart rate (33) and prevents heart rate differences from biasing comparisons. Heart rate variability is therefore reported as the root mean square of the successive differences (RMSSD) divided by the average between-heartbeat intervals (RR).

##### 2.3.2.2 Skin conductance

We used the MentalBioScreen K3 device (Porta Bio Screen GmbH, Berlin, Germany) to measure the skin conductance in microsiemens (µS) on the hypothenar side of the dominant hand (Figure 3B) and the neck, below the hairline (Figure 3C). Using MATLAB® Runtime R2025a (MathWorks, Natick, USA), we calculated the mean SC for each intensity level over the last two minutes of the 3-minute recordings, to account for delayed physiological responses and the participant's initial nervousness.

#### 2.3.3 Movement-based measures

##### 2.3.3.1 Activity counts

We used a Shimmer3® IMU (Shimmer Research Ltd, Dublin, Ireland) to assess the activity counts of each intensity level. We attached the sensor dorsally around the wrist of the participant's dominant hand for the mental condition and on the non-dominant hand for the motor condition (Figure 3D). Using an open-source script, we computed the activity counts per minute (AC/min) (34). We used the AC/min output from the 30 s motor calibration test as the maximal AC/min capacity (ACmax). By normalizing each intensity level's output to the maximal capacity, we calculated the percentage of the maximum AC capacity (%ACmax).

##### 2.3.3.2 Movement repetitions

We added the number of successful and unsuccessful interactions from the exergame's output to derive the total number of MOV the participants performed. We defined the maximal movement repetition capacity (MOVmax) as the total number of MOV from the 30-second motor calibration test. We used it to normalize each intensity level's output, getting the percentage of the maximum number of movement repetitions capacity (%MOVmax).

Some participants surpassed the 100% capacity threshold of %ACmax and %MOVmax for the “very difficult” intensity level. For a more natural visual representation and interpretation of the results, we scaled the data to 100%.

#### 2.3.4 Self-reported measure: NASA-TLX questionnaire

We used the German version of the NASA-TLX questionnaire for children (25), which evaluates workload over six dimensions: mental demand, physical demand, temporal demand, performance, effort, and frustration. To address numerical literacy concerns, the participants used a numberless LEGO® scale. For the analysis, we examined the overall score alongside the mental, physical, and effort subscale scores. We chose these specific subscales in addition to the overall score because we expected the mental subscale to provide insights into increases in cognitive load, the physical subscale to give insights into increases in motor load, and the effort subscale to act as a composite measure.

A more detailed explanation of the protocol, the exergames, and the data processing of each of the measures can be found in Goikoetxea-Sotelo & Van Hedel (12).

### 2.4 Statistical analysis

We analyzed the data using R 4.3.2 (RStudio Inc., Boston, USA). We used the mean values of the two measurements for each condition and intensity level, summarized the data using descriptive statistics, and checked for normality with the Shapiro–Wilk test. We performed separate statistical analyses for each load type. We applied a one-way repeated measures ANOVA for normally distributed data and a Friedman's test for non-normally distributed data to assess statistical differences among the three intensity levels. We set the alpha level at 0.05. In the case of finding significant differences, we performed the necessary *post-hoc* test, i.e., a t-test for normally distributed data and a Wilcoxon signed-rank test for non-normally distributed data. We included the Bonferroni correction for multiple comparisons. In addition, we also computed effect sizes for each paired comparison; Cohen's d (0.2 = small, 0.5 = medium, and 0.8 = large) in case of parametric data and Wilcoxon's r (0.1 = small, 0.3 = medium, and 0.5 = large) in case of non-parametric data.

To assess the test-retest reliability of each measure, we computed the ICCs. In line with Koo and Li (35), we applied a two-way mixed effect, absolute agreement, single rater/measurement ICC form, equivalent to the ICC (2,1) model proposed by Shrout & Fleiss (36) and reported the 95% confidence intervals. We classified ICC values below 0.50 as poor reliability, 0.50–0.75 as moderate, 0.75–0.90 as good, and above 0.90 as excellent.

## 3 Results

Thirty children and adolescents (19 females), with a mean age of 13.3 years (SD 2.8; range 9–19 years), participated in the study. Fourteen participants were diagnosed with cerebral palsy, eight with traumatic brain injury, four with stroke, two with a Pilocytic Astrocytoma, one with Guillan-Barré, and one with meningoencephalitis. Seventeen children predominantly trained their right arm, while thirteen predominantly trained their left arm. Data loss of intensity measures occurred due to malfunctioning of the systems or due to bad data quality. If the data loss occurred in only one session, we used the data for the comparison analysis but not for the reliability analysis (the number of datasets included for the analyses can be found in Tables 1–3).

**Table 1:** Responses of success rate and the intensity measures to mental load.

Measure (unit)	*N*	Mental condition										
		Median (IQR[Q1,Q3)			primary test		*Post-Hoc* tests (Comparison level)					
		Very easy	Challenging	Very difficult	Statistic	*P*-value	Very easy—challenging		Very easy—very difficult		Challenging—very difficult	
							*P*-value	Effect size	*P*-value	Effect size	*P*-value	Effect size
Success rate (%)	30	82 [76, 86]	62 [53, 74]	46 [37, 54]	134.61^a^	**<0.001**	**<0.001**	1.72	**<0.001**	2.97	**<0.001**	1.32
HRV (RMSSD/RR)	27	0.049 [0.029, 0.055]	0.041 [0.030, 0.053]	0.046 [0.029, 0.054]	0.35^a^	0.71	**–**	**–**	**–**	**–**	**–**	**–**
SC Hand (µS)	26	5.4 [3, 8.6]	5.1 [3.1, 9.3]	6.0 [3.0, 9.5]	3.69^b^	0.16	**–**	**–**	**–**	**–**	**–**	**–**
SC Neck (µS)	26	1.9 [1.2, 3.3]	2.0 [1.3, 3.3]	2.1 [1.3, 3.1]	1^b^	0.61	**–**	**–**	**–**	**–**	**–**	**–**
%ACmax (%)	28	0.7 [0.5, 1.1]	1 [0.6, 1.7]	0.7 [0.5, 1.3]	4.36^b^	0.11	**–**	**–**	**–**	**–**	**–**	**–**
%MOVmax (%)	30	12 [10, 15]	10 [8, 12]	6 [5, 9]	52.45^b^	**<0.001**	**<0.001**	0.86	**<0.001**	0.87	**<0.001**	0.82
NASA-TLX (#)	28	230 [174, 267]	276 [241, 323]	350 [304, 427]	146.37^a^	**<0.001**	**<0.001**	0.87	**<0.001**	1.59	**<0.001**	1.07
NASA-TLX mental (#)	28	54 [30, 68]	64 [48, 73]	78 [70, 90]	20.19^a^	**<0.001**	**0.03**	0.54	**<0.001**	1.03	**<0.001**	0.80
NASA-TLX physical (#)	28	40 [19, 50]	45 [20, 53]	50 [39, 73]	14.63^b^	**<0.001**	0.28	0.32	**0.001**	0.70	**0.05**	0.43
NASA-TLX effort (#)	28	44 [29, 51]	50 [45, 58]	68 [58, 83]	28.10^a^	**<0.001**	**0.002**	0.73	**<0.001**	1.19	**<0.001**	0.85

^a^ Parametric test (ANOVA & Cohen's d).

^b^ Non-parametric test (Friedman & Wilcoxon's R). Effect sizes are reported as absolute magnitudes to ensure consistency across parametric and nonparametric tests and to avoid confusion regarding the direction of group differences. Effect size cut offs are 0.2 = small, 0.5 = medium, and 0.8 = large for Cohen's d and 0.1 = small, 0.3 = medium, and 0.5 = large for Wilcoxon's r.

IQR, inter quantile range; Q1, first quantile; Q3, third quantile; HRV, heart rate variability; RMSSD, root mean square of successive differences; RR, r-to-r ratio; SC, skin conductance; %ACmax, number of activity counts, expressed as a percentage of maximum capacity; %MOVmax, number of movement repetitions, expressed as a percentage of maximum capacity; NASA-TLX, national aeronautics and space administration—task load index.

Bold type indicates statistical significance.

**Table 2:** Responses of success rate and the intensity measures to motor load.

Measure (unit)		Motor condition										
	*N*	Median (IQR[Q1,Q3)			primary test		*Post-Hoc* tests (Comparison level)					
		Very easy	Challenging	Very difficult	Statistic (F or *χ*^2^)	*P*-value	Very easy—challenging		Very easy—very difficult		Challenging—very difficult	
							*P*-value	Effect size	*P*-value	Effect size	*P*-value	Effect size
Success Rate (%)	30	99 [99, 100]	90 [81, 94]	53 [41, 65]	60^b^	**<0.001**	**<0.001**	0.87	**<0.001**	0.87	**<0.001**	0.87
HRV (RMSSD/RR)	27	0.044 [0.028, 0.060]	0.034 [0.021, 0.049]	0.027 [0.020, 0.046]	29.63^b^	**<0.001**	**<0.001**	0.70	**<0.001**	0.84	**<0.001**	0.76
SC Hand (µS)	26	8.3 [5.2, 12.1]	8.8 [6.2, 12.9]	9.4 [6.4, 13.0]	2.84^b^	0.24	**–**	**–**	**–**	**–**	**–**	**–**
SC Neck (µS)	26	2.0 [1.1, 3.7]	1.9 [1.1, 3.8]	1.7 [1.1, 3.3]	0.23^b^	0.89	**–**	**–**	**–**	**–**	**–**	**–**
%ACmax (%)	28	66 [56, 72]	79 [74, 86]	98 [92, 100]	38^b^	**<0.001**	**<0.001**	0.70	**<0.001**	0.81	**0.001**	0.80
%MOVmax (%)	30	31 [30, 31]	70 [69, 71]	94 [89, 97]	60^b^	**<0.001**	**<0.001**	0.87	**<0.001**	0.87	**<0.001**	0.87
NASA-TLX (#)	28	136 [97, 203]	316 [254, 373]	433 [380, 488]	147.37^a^	**<0.001**	**<0.001**	1.88	**<0.001**	2.63	**<0.001**	1.88
NASA-TLX mental (#)	28	20 [6, 36]	48 [29,80]	63 [43, 83]	40.66^b^	**<0.001**	**0.001**	0.80	**<0.001**	0.74	**0.004**	0.63
NASA-TLX physical (#)	28	50 [29, 65]	80 [50, 88]	93 [78, 98]	42.72^b^	**<0.001**	**<0.001**	0.76	**<0.001**	0.87	**<0.001**	0.79
NASA-TLX effort (#)	28	23 [14, 43]	68 [50, 83]	91 [75, 98]	43.51^b^	**<0.001**	**<0.001**	0.86	**<0.001**	0.87	**<0.001**	0.74

^a^ Parametric test (ANOVA & Cohen's d).

^b^ Non-parametric test (Friedman & Wilcoxon's R). Effect sizes are reported as absolute magnitudes to ensure consistency across parametric and nonparametric tests and to avoid confusion regarding the direction of group differences. Effect size cut offs are 0.2 = small, 0.5 = medium, and 0.8 = large for Cohen's d and 0.1 = small, 0.3 = medium, and 0.5 = large for Wilcoxon's r.

IQR, inter quantile range; Q1, first quantile; Q3, third quantile; HRV, heart rate variability; RMSSD, root mean square of successive differences; RR, r-to-r ratio; SC, skin conductance; %ACmax, number of activity counts, expressed as a percentage of maximum capacity; %MOVmax, number of movement repetitions, expressed as a percentage of maximum capacity; NASA-TLX, national aeronautics and space administration—task load index.

Bold type indicates statistical significance.

**Table 3:** Reliability of the success rate and the intensity measures.

Measure	*N*	ICC (95% CI [LL,UL)											
		Mental condition						Motor condition					
		Very easy	*P*-value	Challenging	*P*-value	Very difficult	*P*-value	Very easy	*P*-value	Challenging	*P*-value	Very difficult	*P*-value
Success Rate	30	0.33 [−0.03, 0.61]	**0.036**	0.60 [0.31, 79]	**0.001**	0.22 [−0.14, 53]	0.12	0.62 [0.35, 0.80]	**<0.001**	0.90 [0.79, 0.95]	**<0.001**	0.66 [0.39, 0.83]	**<0.001**
HRV	26	0.81 [0.63, 0.91]	**<0.001**	0.81 [0.62, 0.91]	**<0.001**	0.84 [0.67, 0.92]	**<0.001**	0.63 [0.33, 0.81]	**<0.001**	0.56 [0.23, 0.77]	**0.01**	0.79 [0.59, 0.90]	**<0.001**
SC Hand	23	0.61 [0.28, 0.81]	**<0.001**	0.63 [0.31, 0.82]	**<0.001**	0.57 [0.22, 0.79]	**0.001**	0.81 [0.61, 0.92]	**<0.001**	0.70 [0.42, 0.86]	**<0.001**	0.57 [0.22, 0.79]	**<0.001**
SC Neck	23	0.58 [0.24, 0.80]	**<0.001**	0.74 [0.49, 0.88]	**<0.001**	0.50 [0.13, 0.75]	**0.003**	0.72 [0.46, 0.87]	**<0.001**	0.83 [0.63, 0.92]	**<0.001**	0.69 [0.41, 0.86]	**<0.001**
%ACmax	27	0.45 [0.10, 0.70]	**0.007**	0.42 [0.07, 0.68]	**0.011**	0.63 [0.34, 0.81]	**<0.001**	0.87 [0.74, 0.94]	**<0.001**	0.79 [0.60, 0.90]	**<0.001**	0.71 [0.47, 0.86]	**<0.001**
%MOVmax	30	0.85 [0.69, 0.93]	**<0.001**	0.76 [0.56, 0.88]	**<0.001**	0.66 [0.41, 0.82]	**<0.001**	0.32 [−0.02, 0.60]	**0.034**	0.34 [0.01, 0.61]	**0.02**	0.50 [0.18, 0.72]	**0.002**
NASA-TLX	28	0.63 [0.35, 0.81]	**<0.001**	0.46 [0.12, 0.70]	**0.005**	0.37 [0.01, 0.65]	**0.02**	0.64 [0.36, 0.82]	**<0.001**	0.72 [0.45, 0.87]	**<0.001**	0.64 [0.36, 0.81]	**<0.001**
NASA-TLX (mental)	28	0.75 [0.53, 0.87]	**<0.001**	0.50 [0.16, 0.73]	**0.003**	0.67 [0.41, 0.83]	**<0.001**	0.50 [0.17, 0.73]	**0.003**	0.77 [0.57, 0.89]	**<0.001**	0.55 [0.24, 0.76]	**<0.001**
NASA-TLX (physical)	28	0.66 [0.38, 0.82]	**<0.001**	0.48 [0.15, 0.72]	**0.004**	0.54 [0.36, 0.81]	**<0.001**	0.49 [0.14, 0.73]	**0.001**	0.62 [0.33, 0.80]	**<0.001**	0.30 [−0.035, 0.59]	**0.038**
NASA-TLX (effort)	28	0.24 [−0.11, 0.55]	0.09	0.00 [−0.30, 0.33]	0.5	0.45 [0.11, 0.70]	**0.007**	0.36 [0.01, 0.64]	**0.02**	0.71 [0.47, 0.85]	**<0.001**	0.83 [0.66, 0.92]	**<0.001**

ICC, intraclass correlation coefficient; LL, lower limit; UL, upper limit; HRV, heart rate variability; SC, skin conductance; %ACmax, number of activity counts, expressed as a percentage of maximum capacity; %MOVmax, number of movement repetitions, expressed as a percentage of maximum capacity; NASA-TLX, national aeronautics and space administration—task load index.

Bold type indicates statistical significance.

**Figure 4:**
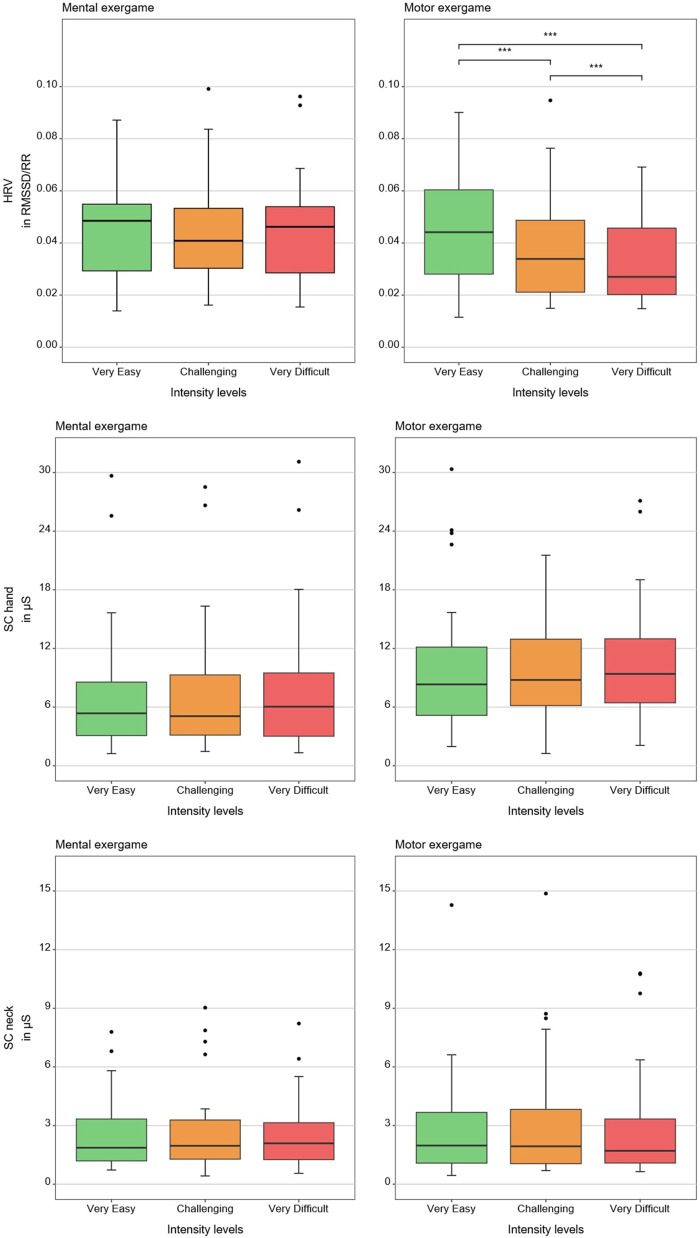
Results of the physiological measures. The boxplots reflect the median and interquartile ranges. The stars indicate the significance level of the paired comparisons. * = *p* ≤ 0.05. ** = *p* < 0.01. *** = *p* < 0.001.

**Figure 5:**
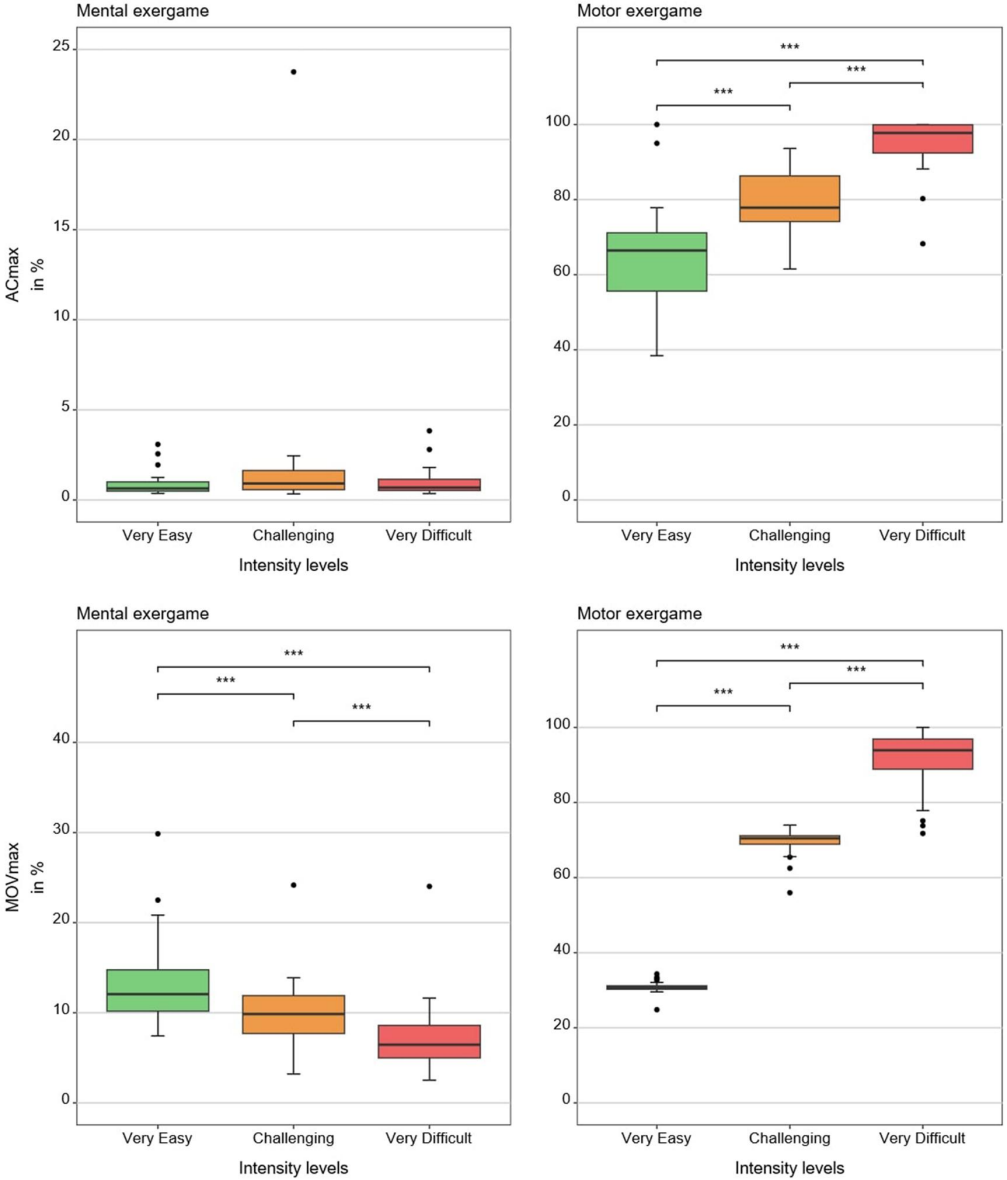
Results of the movement-based measures. The boxplots reflect the median and interquartile ranges. The stars indicate the significance level of the paired comparisons. * = *p* ≤ 0.05. ** = *p* < 0.01. *** = *p* < 0.001.

**Figure 6:**
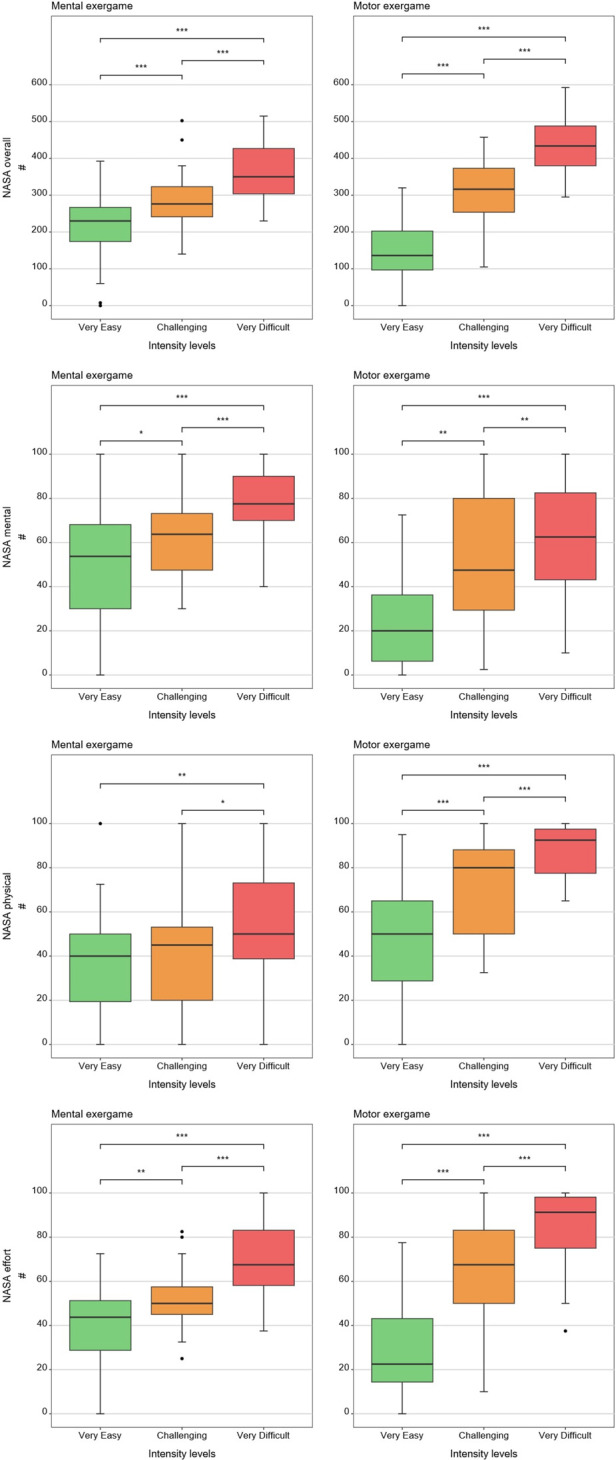
Results of the patient-reported measures. The boxplots reflect the median and interquartile ranges. The stars indicate the significance level of the paired comparisons. * = *p* ≤ 0.05. ** = *p* < 0.01. *** = *p* < 0.001.

Tables 1, 2 show medians and interquartile ranges, the primary statistic tests, and *post-hoc* comparisons with effect sizes. Percentage MOVmax, and the NASA-TLX overall score and its mental and effort subscales showed statistically significant differences across the three therapy intensity levels for both conditions. Heart rate variability, %ACmax, and the NASA-TLX physical dimension showed statistically significant differences across the three therapy intensity levels for the motor condition but not for the mental condition. Skin conductance did not show statistically significant differences across therapy intensity levels for any condition (see Tables 1, 2; Figures 4–6).

Concerning test-retest reliability, Table 3 reports ICCs with 95% confidence intervals and *p*-values testing whether ICCs are greater than zero. Overall, the test-retest reliability ranged from poor (ICC = 0.00 [−0.30, 0.33) for the NASA-TLX effort subscale at the “challenging” mental intensity level to good (ICC = 0.87 [0.74, 0.94) for the %ACmax at the “very easy” intensity level of the motor condition (see Table 3).

## 4 Discussion

This study is the first to explore how different candidate intensity measures respond to changes in mental and motor intensity during an upper limb exergame in children with neurological diagnoses. We also evaluated the test-retest reliability of these measures across two independent test sessions. The results suggest that the candidate intensity measures respond differently depending on the type of load and intensity level. The main findings were: first, the NASA-TLX overall score and its mental and effort subscales, and the %MOVmax responded to increases in mental and motor load. Second, HRV, %ACmax, and the NASA-TLX physical subscale responded to increases in motor load but not, or only partially, to increases in mental load. Third, SC did not respond to increases in any type of load. The reliability of the measures varied widely. Heart rate variability for the mental condition was the only measure showing ICC scores higher than the acceptance threshold (ICC > 0.75) for each of the intensity levels, closely followed by %ACmax for the motor condition, which reached acceptance for the “very easy” and “challenging” intensity levels, and remained near to the acceptance threshold for the “very difficult” intensity level (ICC = 0.71).

### 4.1 Response

The success rate, serving as our control variable, responded to all intensity levels across each load type, confirming the suitability of our task difficulty settings. Additionally, the success rate percentages closely aligned with our targets, indicating that the games were properly designed and possess a valid construct (12).

Heart rate variability decreased with increasing motor load, confirming our hypothesis and in line with previous findings in typically developing children (12). Contrary to our hypothesis and unlike prior work reporting HRV responses to mental load (e.g., chess, driving) (37–40), we found no effect, likely because our task involved a simple visual search task, insufficient to elicit central autonomic responses despite higher perceived load.

Contrary to our hypothesis, SC did not react to increasing load at any level. Although we found similar results in a previous study (12), these contrast with existing literature showing SC responses to increasing task difficulty during video gaming (21, 22, 41, 42). The discrepancy may reflect populational, procedural, and technological differences or that SC is less suited for pediatric neurorehabilitation tasks.

As hypothesized, %ACmax and %MOVmax increased at higher motor intensity levels, which is in line with our previous studies (12, 23). Contrary to what we saw in Goikoetxea-Sotelo & Van Hedel (12), %ACmax showed no response to increasing mental load, suggesting that our mouse-based control succeeded in equalizing movement across mental load levels. Still, the NASA-TLX physical dimension showed some increase under higher mental load, perhaps because shorter movement execution times made the motor action more demanding.

In line with our hypothesis, the NASA-TLX overall scores and subscales responded to both load levels. This extends prior findings in healthy children (25) by showing its ability to respond to three mental and three motor intensity levels in children with neurological diagnoses. The effort subscale, in particular, tracked overall exercise intensity and correlated with the NASA-TLX closely (*r* = 0.86 for the “very easy”, *r* = 0.58 for the “challenging”, and *r* = 0.70 for the “very difficult” mental condition, and *r* = 0.81 for the “very easy”, *r* = 0.79 for the “challenging”, and *r* = 0.77 for the “very difficult” motor condition), suggesting it may serve as a simplified alternative to the full questionnaire.

### 4.2 Reliability

Heart rate variability showed the most consistent test–retest reliability, with values generally in the moderate-to-good range. This aligns with prior work in clinical and healthy populations in different conditions (e.g., exergaming, light exercise and rest) (23, 43–46).

Skin conductance showed moderate to good test-retest reliability, which is in line with its variable performance reported across different experimental contexts (23, 47–49).

Percentage ACmax demonstrated moderate-to-good reliability under motor conditions, which is in line with the literature (23, 50, 51), but poor reliability under mental conditions, which is unsurprising, as it was not designed to quantify mental load. Percentage MOVmax showed poor to moderate reliability. As it is known that normalization minimizes between-participant data variability, reducing ICC values (52), we repeated the analysis in non-normalized data, which produced excellent ICC scores (ICC = 0.90 for the “very easy”, ICC = 0.89 for the “challenging”, and ICC = 0.91 for the “very difficult” motor intensity levels), similar to those in our previous study (23).

Last, the NASA-TLX overall and subscales demonstrated variable reliability, from poor to moderate depending on the load type and dimension. These findings are consistent with prior reports of unstable NASA-TLX reliability in typically developing adults (53, 54) and older adults with cognitive impairments (55).

### 4.3 Potential for clinical applicability

Heart rate variability responded to motor load, suggesting potential for differentiating motor intensity levels. It is easy to use and could, in principle, be applied in therapy as heart rate is in cardiovascular training. However, its relationship to a participant's individual capacity remains unclear, and reliability exceeded acceptable thresholds only at the highest intensity, limiting clinical applicability.

Skin conductance did not respond to changes in motor or mental intensity and therefore appears unsuitable for quantifying intensity in upper limb neurorehabilitation.

Percentage ACmax and %MOVmax reacted to increasing motor load and exhibited good reliability (the latter in its non-normalized version, i.e., MOV/min), showing their potential as intensity measures for motor tasks. Yet their broader use is limited by practical constraints: 1) counting movement repetitions, especially in conventional therapy, is time-consuming, maximal capacity testing is rarely feasible, and intensity can only be calculated retrospectively, preventing real-time adjustments. From a clinical standpoint, AC/min may be the most feasible option, as it provides a session-level indicator and allows comparisons between therapies with similar goals.

The NASA-TLX and its subscales differentiated between most mental and motor load levels. Furthermore, the overall and effort scores showed strong correlations, suggesting the effort subscale could serve as a simplified alternative. This is especially relevant for participants with cognitive impairments, for whom the full questionnaire may be too complex. However, reliability was inconsistent and rarely reached acceptable thresholds. Moreover, its subjective and relative nature, together with inter-individual variability, complicates the definition of standardized workload thresholds (16, 56) and does not enable ongoing measurements. Thus, while useful for rapid estimation, NASA-TLX should be interpreted with caution.

### 4.4 Methodological considerations

The novelty of the games may have influenced reliability. Early familiarization and skill acquisition could have led patients to perceive and respond differently across sessions, especially in the mental condition, where search strategies develop over time. Allowing a longer familiarization period, or advance practice, could help participants adapt and yield more consistent responses.

Our decision to perform maximal capacity tests at each session minimized the effect of confounding factors such as learning effects or daily form but might have affected test-retest reliability estimates.

Introducing the mouse as a controller in the mental condition reduced unintended arm movements but introduced its own motor demands. Alternative input methods such as eye tracking may isolate mental load more effectively.

### 4.5 Limitations

As a single center study, selection bias cannot be ruled out. Our institution is a public inpatient rehabilitation center that treats children from across Switzerland with diverse diagnoses and severity levels. While inpatient settings typically include more severely affected patients than outpatient centers, we expect the broad spectrum of participants to help reduce the risk of systematic bias.

We did not normalize physiological data to each participant's baseline, which might have reduced individual variability and day-to-day fluctuations. Although resting measures were collected, they often exceeded values from the “very difficult” condition, likely due to initial nervousness or the effort required for some children to reach the testing room.

Another limitation is the use of the NASA-TLX in a pediatric neurological sample. While prior work supports its validity even in small children (25) and our findings support its usage at group level, individual-level responses in this population may be less robust.

A further limitation is the exclusive use of mean skin conductance as the electrodermal activity marker. Although chosen for consistency and feasibility, mean SC is sensitive to baseline drifts and may be less responsive to short-term workload changes than phasic electrodermal activity indices. Using alternative or complementary phasic markers might have revealed different patterns of results.

### 4.6 Future research

Future research should explore methods to minimize familiarization effects, such as providing longer training or advance practice with exergames. Alternative input methods like eye tracking may allow cleaner separation of mental and motor load. Simplified and feasible metrics such as AC/min and MOV/min warrant further validation in different therapy contexts, while the role of subjective measures such as the NASA-TLX effort subscale should be clarified in larger and more diverse populations. Finally, studies across multiple centers will be needed to confirm generalizability and support the development of standardized intensity measures for clinical practice.

## 5 Conclusion

In rehabilitation, accurately measuring therapy intensity is critical. In this study, we identified some candidate intensity measures that responded to increases in mental and motor intensity, suggesting they could be used to compare the intensity of therapies. However, the reliability of the measures often fell below the “acceptable” threshold. Furthermore, identifying measures that enable ongoing adaption of therapy intensity remains challenging, especially because we still do not know how the output of some of the measures reflects the participant's capacity. Before drawing definitive conclusions, future studies should analyze how controlled increases in motor and mental load affect the responses in clinically relevant therapeutic settings.

## Data Availability

The raw data supporting the conclusions of this article will be made available by the authors, without undue reservation.

## References

[1] Metzler MJ Haspels E Brunton L Andersen J Pritchard L Herrero M Goals of children with unilateral cerebral palsy in a brain stimulation arm rehabilitation trial. Dev Med Child Neurol. (2021) 63(5):584–91. 10.1111/dmcn.14763 33368181

[2] Rast FM Labruyère R. ICF mobility and self-care goals of children in inpatient rehabilitation. Dev Med Child Neurol. (2020) 62(4):483–8. 10.1111/dmcn.14471 31984500

[3] Krakauer JW. Motor learning: its relevance to stroke recovery and neurorehabilitation. Curr Opin Neurol. (2006) 19(1):84–90. 10.1097/01.wco.0000200544.29915.cc 16415682

[4] Kleim JA Jones TA. Principles of experience-dependent neural plasticity: implications for rehabilitation after brain damage. J Speech Lang Hear Res. (2008) 51(1):225–39. 10.1044/1092-4388(2008/018) 18230848

[5] Page SJ Schmid A Harris JE. Optimizing terminology for stroke motor rehabilitation: recommendations from the American congress of rehabilitation medicine stroke movement interventions subcommittee. Arch Phys Med Rehabil. (2012) 93(8):1395–9. 10.1016/j.apmr.2012.03.005 22446292 PMC3413452

[6] Goikoetxea-Sotelo G van Hedel HJA. Defining, quantifying, and reporting intensity, dose, and dosage of neurorehabilitative interventions focusing on motor outcomes. Front Rehabil Sci. (2023) 4:1139251. 10.3389/fresc.2023.1139251 37637933 PMC10457006

[7] Selye H. Stress and the general adaptation syndrome. Br Med J. (1950) 1(4667):1383–92. 10.1136/bmj.1.4667.1383 15426759 PMC2038162

[8] Turner A. The science and practice of periodization: a brief review. Strength Cond J. (2011) 33(1):34–46. 10.1519/SSC.0b013e3182079cdf.

[9] Kaur G English C Hillier S. How physically active are people with stroke in physiotherapy sessions aimed at improving motor function? A systematic review. Stroke Res Treat. (2012) 2012:820673. 10.1155/2012/820673 22567542 PMC3337516

[10] de Quirós M Douma EH van den Akker-Scheek I Lamoth CJC Maurits NM. Quantification of movement in stroke patients under free living conditions using wearable sensors: a systematic review. Sensors. (2022) 22(3):1050. 10.3390/s22031050 35161796 PMC8840016

[11] Porciuncula F Roto AV Kumar D Davis I Roy S Walsh CJ Wearable movement sensors for rehabilitation: a focused review of technological and clinical advances. PM\&R. (2018) 10(9S2):S220–32. 10.1016/j.pmrj.2018.06.013 30269807 PMC6700726

[12] Goikoetxea-Sotelo G Van Hedel HJA. Responses of candidate intensity measures to different mental and motor load levels using upper limb exergames in typically developing children and adolescents. PLoS One. (2025) 20(6):e0326371. 10.1371/journal.pone.0326371 40561051 PMC12193600

[13] Galy E Paxion J Berthelon C. Measuring mental workload with the NASA-TLX needs to examine each dimension rather than relying on the global score: an example with driving. Ergonomics. (2018) 61(4):517–27. 10.1080/00140139.2017.1369583 28817353

[14] DiDomenico A Nussbaum M. Effects of different physical workload parameters on mental workload and performance. Int J Ind Ergon. (2011) 41:255–60. 10.1016/j.ergon.2011.01.008

[15] Eggemeier F Wilson G Kramer A Damos D. Workload Assessment in Multi-task environments. In: Damos DL, editor. Multiple-task Performance. Boca Raton, FL, London and New York, NY: CRC Press, Taylor & Francis Group (2020). p. 207–16. 10.1201/9781003069447-12

[16] Hart SG. Nasa-task load Index (NASA-TLX); 20 years later. Proc Hum Factors Ergon Soc Ann Meeting. (2006) 50(9):904–8. 10.1177/154193120605000909

[17] Said S Gozdzik M Roche TR Braun J Rössler J Kaserer A Validation of the raw national aeronautics and space administration task load index (NASA-TLX) questionnaire to assess perceived workload in patient monitoring tasks: pooled analysis study using mixed models. J Med Internet Res. (2020) 22(9):e19472. 10.2196/19472 32780712 PMC7506540

[18] Boucsein W. Electrodermal activity: second edition. New York, NY: Springer (2012). 10.1007/978-1-4614-1126-0

[19] Malik M Bigger JT Camm AJ Kleiger RE Malliani A Moss AJ Heart rate variability: standards of measurement, physiological interpretation, and clinical use. Eur Heart J. (1996) 17(3):354–81. 10.1093/oxfordjournals.eurheartj.a014868 8737210

[20] Boettger S Puta C Yeragani VK Donath L Müller H-J Gabriel HHW Heart rate variability, QT variability, and electrodermal activity during exercise. Med Sci Sports Exercise. (2010) 42(3):443–8. 10.1249/MSS.0b013e3181b64db1 19952826

[21] Tian Y Bian Y Han P Wang P Gao F Chen Y. Physiological signal analysis for evaluating flow during playing of computer games of varying difficulty. Front Psychol. (2017) 8:1121. 10.3389/fpsyg.2017.01121 28725206 PMC5495833

[22] Wiberg H Nilsson E Lindén P Svanberg B Poom L. Physiological responses related to moderate mental load during car driving in field conditions. Biol Psychol. (2015) 108:115–25. 10.1016/j.biopsycho.2015.03.017 25857673

[23] Goikoetxea-Sotelo G Van Hedel HJA. Responses of several measures to different intensity levels of upper limb exergames in children with neurological diagnoses: a pilot study. Front Rehabil Sci. (2024) 5:1405304. 10.3389/fresc.2024.1405304 39507588 PMC11538011

[24] Mokkink LB Terwee CB Patrick DL Alonso J Stratford PW Knol DL The COSMIN checklist for assessing the methodological quality of studies on measurement properties of health status measurement instruments: an international delphi study. Qual Life Res. (2010) 19(4):539–49. 10.1007/s11136-010-9606-8 20169472 PMC2852520

[25] Laurie-Rose C Frey M Ennis A Zamary A. Measuring perceived mental workload in children. Am J Psychol. (2014) 127(1):107–25. 10.5406/amerjpsyc.127.1.0107 24720100

[26] Alcantara JMA Plaza-Florido A Amaro-Gahete FJ Acosta FM Migueles JH Molina-Garcia P Impact of using different levels of threshold-based artefact correction on the quantification of heart rate variability in three independent human cohorts. J Clin Med. (2020) 9(2):325. 10.3390/jcm9020325 31979367 PMC7074236

[27] Giles DA Draper N. Heart rate variability during exercise: a comparison of artefact correction methods. J Strength Cond Res. (2018) 32(3):726–35. 10.1519/JSC.0000000000001800 29466273

[28] Rogers B Giles D Draper N Mourot L Gronwald T. Influence of artefact correction and recording device type on the practical application of a non-linear heart rate variability biomarker for aerobic threshold determination. Sensors (Basel). (2021) 21(3):821. 10.3390/s21030821 33530473 PMC7865269

[29] Georgiou K Larentzakis AV Khamis NN Alsuhaibani GI Alaska YA Giallafos EJ. Can wearable devices accurately measure heart rate variability? A systematic review. Folia Med (Plovdiv). (2018) 60(1):7–20. 10.2478/folmed-2018-0012 29668452

[30] Hoffmann B Flatt AA Silva LEV Młyńczak M Baranowski R Dziedzic E A pilot study of the reliability and agreement of heart rate, respiratory rate and short-term heart rate variability in elite modern pentathlon athletes. Diagnostics (Basel). (2020) 10(10):833. 10.3390/diagnostics10100833 33081149 PMC7602793

[31] Sacha J Pluta W. Different methods of heart rate variability analysis reveal different correlations of heart rate variability spectrum with average heart rate. J Electrocardiol. (2005) 38(1):47–53. 10.1016/j.jelectrocard.2004.09.015 15660347

[32] Sacha J Pluta W. Alterations of an average heart rate change heart rate variability due to mathematical reasons. Int J Cardiol. (2008) 128(3):444–7. 10.1016/j.ijcard.2007.06.047 17689709

[33] Sacha J. Why should one normalize heart rate variability with respect to average heart rate. Front Physiol. (2013) 4:306. 10.3389/fphys.2013.00306 24155724 PMC3804770

[34] BrØnd JC Andersen LB Arvidsson D. Generating ActiGraph counts from raw acceleration recorded by an alternative monitor. Med Sci Sports Exercise. (2017) 49(11):2351–60. 10.1249/MSS.0000000000001344 28604558

[35] Koo TK Li MY. A guideline of selecting and reporting intraclass correlation coefficients for reliability research. J Chiropr Med. (2016) 15(2):155–63. 10.1016/j.jcm.2016.02.012 27330520 PMC4913118

[36] Shrout PE Fleiss JL. Intraclass correlations: uses in assessing rater reliability. Psychol Bull. (1979) 86:420–8. 10.1037/0033-2909.86.2.420 18839484

[37] Galy E Cariou M Mélan C. What is the relationship between mental workload factors and cognitive load types? Int J Psychophysiol. (2012) 83(3):269–75. 10.1016/j.ijpsycho.2011.09.023 22008523

[38] Kim H-G Cheon E-J Bai D-S Lee YH Koo B-H. Stress and heart rate variability: a meta-analysis and review of the literature. Psychiatry Investig. (2018) 15(3):235–45. 10.30773/pi.2017.08.17 29486547 PMC5900369

[39] Mukherjee S Yadav R Yung I Zajdel DP Oken BS. Sensitivity to mental effort and test-retest reliability of heart rate variability measures in healthy seniors. Clin Neurophysiol. (2011) 122(10):2059–66. 10.1016/j.clinph.2011.02.032 21459665 PMC3132243

[40] von Rosenberg W Chanwimalueang T Adjei T Jaffer U Goverdovsky V Mandic DP. Resolving ambiguities in the LF/HF ratio: lF-HF scatter plots for the categorization of mental and physical stress from HRV. Front Physiol. (2017) 8:360. 10.3389/fphys.2017.00360 28659811 PMC5469891

[41] Haapalainen E Kim S Forlizzi JF Dey AK. Psycho-physiological measures for assessing cognitive load. Proceedings of the 12th ACM International Conference on Ubiquitous Computing (2010). p. 301–10. 10.1145/1864349.1864395

[42] Ikehara CS Crosby ME. Assessing cognitive load with physiological sensors. 2014 47th Hawaii International Conference on System Sciences (2005) 10. p. 295a. 10.1109/HICSS.2005.103

[43] Ashaie SA Engel S Cherney LR. Test–retest reliability of heart-rate variability metrics in individuals with aphasia. Neuropsychol Rehabil. (2022) 33(4):646–61. 10.1080/09602011.2022.2037438 35179091

[44] Eikeseth FF Sætren SS Benjamin BR Ulltveit-Moe Eikenæs I Sütterlin S Hummelen B. The test-retest reliability of heart rate variability and its association with personality functioning. Front Psychiatry. (2020) 11:1–8. 10.3389/fpsyt.2020.558145 33329098 PMC7672153

[45] Guijt AM Sluiter JK Frings-Dresen MHW. Test-retest reliability of heart rate variability and respiration rate at rest and during light physical activity in normal subjects. Arch Med Res. (2007) 38(1):113–20. 10.1016/j.arcmed.2006.07.009 17174734

[46] Sandercock GRH Bromley PD Brodie DA. The reliability of short-term measurements of heart rate variability. Int J Cardiol. (2005) 103(3):238–47. 10.1016/j.ijcard.2004.09.013 16098384

[47] Cooper SE Dunsmoor JE Koval KA Pino ER Steinman SA. Test–retest reliability of human threat conditioning and generalization across a 1-to-2-week interval. Psychophysiology. (2023) 60(6):e14242. 10.1111/psyp.14242 36546410

[48] Ridderbusch IC Wroblewski A Yang Y Richter J Hollandt M Hamm AO Neural adaptation of cingulate and insular activity during delayed fear extinction: a replicable pattern across assessment sites and repeated measurements. NeuroImage. (2021) 237:118157. 10.1016/j.neuroimage.2021.118157 34020017

[49] Zeidan MA Lebron-Milad K Thompson-Hollands J Im JJY Dougherty DD Holt DJ Test-retest reliability during fear acquisition and fear extinction in humans. CNS Neurosci Ther. (2012) 18(4):313–7. 10.1111/j.1755-5949.2011.00238.x 21592319 PMC6493448

[50] Lakerveld ID. Test-retest Reliability of an IMU Sensor-based method for Measuring Quality Metrics, during the “Reach tot Grasp” Movement in Healthy Adults *(master's thesis).* University of Twente, Enschede. Essay: 89066. (2021). Available online at: https://purl.utwente.nl/essays/89066

[51] O’Keeffe K Argent R Bourke A Shabani S Praestgaard J Muaremi A Test-retest reliability of wireless inertial-sensor derived measurements of knee joint kinematics. 2022 44th Annual International Conference of the IEEE Engineering in Medicine & Biology Society (EMBC) (2022). p. 4218–21. 10.1109/EMBC48229.2022.9871584 36085698

[52] Lee KM Lee J Chung CY Ahn S Sung KH Kim TW Pitfalls and important issues in testing reliability using intraclass correlation coefficients in orthopaedic research. Clin Orthop Surg. (2012) 4(2):149–55. 10.4055/cios.2012.4.2.149 22662301 PMC3360188

[53] Ikuma LH Nussbaum MA Babski-Reeves KL. Reliability of physiological and subjective responses to physical and psychosocial exposures during a simulated manufacturing task. Int J Ind Ergon. (2009) 39(5):813–20. 10.1016/j.ergon.2009.02.005

[54] Xiao Y Wang Z Wang M Lan Y. The appraisal of reliability and validity of subjective workload assessment technique and NASA-task load index. Zhonghua Lao Dong Wei Sheng Zhi Ye Bing Za Zhi. (2005) 23(3):178–81. 16124892

[55] Devos H Gustafson K Ahmadnezhad P Liao K Mahnken JD Brooks WM Psychometric properties of NASA-TLX and index of cognitive activity as measures of cognitive workload in older adults. Brain Sci. (2020) 10(12):994. 10.3390/brainsci10120994 33339224 PMC7766152

[56] Miyake S. Factors influencing mental workload indexes. J UOEH. (1997) 19(4):313–25. 10.7888/juoeh.19.313 9431583

